# Pulvinar and Ventral Thalamic Nuclei Changes Occur Early Along the Psychosis Spectrum

**DOI:** 10.1093/schizbullopen/sgaf016

**Published:** 2025-09-11

**Authors:** Ciaran Browne, Anurag Nasa, Linda Kelly, Sahar Riaz, Vitallia Sooknarine, Michael O’Connor, Orla Mitchell, Emma O’Hora, An Hsu, Ahmad Almulla, Areej Gazzaz, Conan Brady, Colm Healy, Erik O’Hanlon, Michael Connaughton, Mary Cannon, Darren William Roddy

**Affiliations:** Department of Psychiatry, Royal College of Surgeons in Ireland (RCSI) University of Medicine and Health Sciences, Ireland; Department of Psychiatry, Royal College of Surgeons in Ireland (RCSI) University of Medicine and Health Sciences, Ireland; Department of Psychiatry, Royal College of Surgeons in Ireland (RCSI) University of Medicine and Health Sciences, Ireland; Department of Psychiatry, Royal College of Surgeons in Ireland (RCSI) University of Medicine and Health Sciences, Ireland; Department of Psychiatry, Royal College of Surgeons in Ireland (RCSI) University of Medicine and Health Sciences, Ireland; Department of Psychiatry, Royal College of Surgeons in Ireland (RCSI) University of Medicine and Health Sciences, Ireland; Department of Psychiatry, Royal College of Surgeons in Ireland (RCSI) University of Medicine and Health Sciences, Ireland; Department of Psychiatry, Royal College of Surgeons in Ireland (RCSI) University of Medicine and Health Sciences, Ireland; Department of Psychiatry, Royal College of Surgeons in Ireland (RCSI) University of Medicine and Health Sciences, Ireland; Department of Psychiatry, Royal College of Surgeons in Ireland (RCSI) University of Medicine and Health Sciences, Ireland; Department of Psychiatry, Royal College of Surgeons in Ireland (RCSI) University of Medicine and Health Sciences, Ireland; Department of Psychiatry, Royal College of Surgeons in Ireland (RCSI) University of Medicine and Health Sciences, Ireland; Department of Psychiatry, Royal College of Surgeons in Ireland (RCSI) University of Medicine and Health Sciences, Ireland; Department of Psychiatry, Royal College of Surgeons in Ireland (RCSI) University of Medicine and Health Sciences, Ireland; Department of Psychiatry, Royal College of Surgeons in Ireland (RCSI) University of Medicine and Health Sciences, Ireland; Department of Psychiatry, Royal College of Surgeons in Ireland (RCSI) University of Medicine and Health Sciences, Ireland; Department of Psychiatry, Royal College of Surgeons in Ireland (RCSI) University of Medicine and Health Sciences, Ireland

**Keywords:** psychotic experiences, thalamic nuclei, MRI, neurobiological markers, psychosis, adolescents, pulvinar, ventral thalamus

## Abstract

**Background:**

Psychosis may be conceptualized as a spectrum disorder, with psychotic experiences (PEs), fleeting, subtle symptoms not warranting clinical presentation—at its mildest end. The thalamus, particularly its pulvinar region, is implicated in coordinating cortical synchrony and attention, and may contribute to psychosis. Reduced pulvinar volumes have been observed in severe psychosis and in individuals lower on the spectrum. This study examines thalamic nuclei changes in young adolescents with PEs.

**Methods:**

A community-based sample of 95 adolescents aged 11-13 years (53 with PEs, 42 healthy controls) underwent magnetic resonance imaging (MRI), with thalamic nuclei volumes calculated using Freesurfer. Magnetic resonance imaging (MRI) was repeated at 2- and 5-year follow-up. Analyses of covariance and linear mixed-effects (LME) models assessed group-wise differences at each timepoint (TP) and longitudinally. Individual nuclei were recombined into anatomical composites (eg, pulvinar, ventral) for targeted analysis.

**Results:**

Compared with controls, adolescents with PEs had significantly smaller left pulvinar composite volumes at TP2 (*P* = .01) and TP3 (*P* = .019). Linear mixed-effects revealed a significant longitudinal reduction in left pulvinar volume (*P* = .008, false discovery rate [FDR]-corrected) and a significant increase in left ventral volumes (*P* = .013, FDR-corrected).

**Conclusions:**

Thalamic changes linked to higher-risk psychotic states appear detectable in nonclinical adolescents with subthreshold PEs. This divergence from normative developmental trajectories may indicate early alterations in neural circuits governing attention, cortical synchrony, and dopaminergic function. Identifying such early deviations could refine our understanding of psychosis vulnerability. Replication in larger, more diverse cohorts is warranted to confirm these preliminary findings and assess their predictive value.

## Introduction

Psychosis is a complex psychiatric syndrome characterized by impairments in reality testing, which lead to hallucinations, delusions, and abnormal behaviors.^1^ Psychosis is thought to occur along a spectrum with diagnosable psychotic disorders (eg, chronic schizophrenia) at the severe end and varying degrees of less intense symptoms and impairment down along the spectrum, such as schizotypy and prodromal/attenuated symptoms, with strict boundaries across the spectrum often unclear and fluid.^2^ Phenomena known as psychotic experiences (PEs) could be considered at the lowest end of this spectrum involving mild symptoms of impaired reality testing, like fleeting hallucinations and delusions, often without leading individuals to seek professional healthcare.^3^

Psychotic experiences are more common than clinically diagnosed psychotic disorders, with a meta-analysis estimating their prevalence at around 5% in the general population,^4^ with the rates of PEs in adolescents considered to be higher.^5^ The incidence of these subthreshold symptoms increases with age between 13 and 24, peaking during late adolescence^6^ and declining into adulthood. Having previously reported a PE is linked to a 4-fold increased risk of developing a diagnosable psychotic disorder such as schizophrenia^5^ as well as an increased risk of depressive symptoms, a lower quality of life,^7^ impaired social and role functioning,^8^ suicidal feelings or self-harm behaviors,^9^ and physical symptoms^10^ in later life.

The neurobiology of psychosis is complex with often diffuse psychopathology (problems in reality testing, cognition, behavior, insight, and motor difficulties) reflected in widespread brain involvement including the frontal networks^11^^,^^12^ limbic system,^13^ subcortical regions,^14^ cerebellum,^15^ and motor areas.^16^ Brain changes have been found along the length of the psychosis spectrum from PEs^17–19^ to chronic schizophrenia.^20^^,^^21^ Dyssynchrony between brain regions (ie, impairments in temporal coordination across brain networks)^22^ has been suggested as an explanation for this diffuse psychopathology and brain involvement.^23^ A key structure coordinating information from the external–internal environment and facilitating cortical synchronization is the thalamus, suggesting a central role for this relay region in psychosis.^24^

The thalamus is a bilateral egg-shaped structure above the midbrain and integrates sensory and cortically derived information through relaying neurons that spread outwards to the cortex in all directions.^25^ This allows an exchange, integration, and processing of motor, sensory, cortical, and subcortical information and external–internal world communication. As such the thalamus is thought to be intimately involved in consciousness and alertness.^26^ The thalamus can be divided into smaller subunits, called nuclei.^27^ These nuclei may be reorganized into 7 anatomico-functional groups (the anterior, lateral, ventral, intralaminar, medial, posterior, and reticular groups^27^ based on related connectivity and functions (Table S1). Until recently, in-vivo human studies of the thalamus were largely limited to lesion^28^ or post-mortem studies.^29^ Advancements in magnetic resonance imaging (MRI) acquisition techniques and thalamic image processing, like Freesurfer 7.0, have enabled more precise investigation of individual thalamic nuclei, expanding beyond traditional studies focused on overall thalamic structure and function.

Thalamic volume changes have been found along the psychosis spectrum from schizophrenia^30^ to those at high familial risk of psychosis. Manual subunit parcellation and automatic parcellation techniques consistently uncover a smaller thalamic pulvinar region across the spectrum, including schizophrenia,^31^ first episode psychosis,^32^ and in young people with psychotic spectrum symptoms.^33^ The pulvinar region is integral to visual processing and general cognitive function,^34^ in particular goal-directed attention and cognitive flexibility. It also has a role in coordinating cortical synchrony.^35^ Other nuclei potentially involved in psychosis, include those of the ventral^33^ and medial^36^ groups.

The thalamus undergoes key structural changes during adolescence, a period marked by refinement of neural circuits supporting cognition and perception.^37^ Typically, thalamic volume increases in late childhood, stabilizes in early adolescence, and gradually declines into adulthood. The pulvinar, involved in attention and cortical synchrony, tends to maintain or increase in volume during this stage, reflecting the maturation of higher-order cognitive functions.^38^ Deviations from these normative trajectories may indicate early neurodevelopmental disruptions relevant to psychosis risk.

This study investigates whether adolescents with PEs, who do not meet diagnostic thresholds, show atypical developmental trajectories in specific thalamic nuclei. Given the roles of the pulvinar and ventral groups in attention, sensory integration, and thalamocortical connectivity, we hypothesize that adolescents with PEs will show volumetric differences in these nuclei compared to typically developing peers. Using high-resolution MRI and Freesurfer 7.0, we examined thalamic nuclei volumes in a school-based sample assessed at 3 time points over 5 years.

## Methods

### Participants

Participants were recruited from the Adolescent Brain Development (ABD) project.^39^ Between July 2007 and September 2010, 212 young people aged between 11 and 13 years were recruited from primary schools in Dublin and Kildare, Ireland. All 212 participants were Caucasian and native English-speaking. A subsample of 95 of these young people also agreed to take part in a neuroimaging study. An a-priori power analysis (G*Power v3.1) was conducted to determine the required sample size for detecting a medium effect size (*f* = 0.25) with 80% power. These participants were subsequently followed through their adolescence. All participants were assessed, as part of the ABD project, using a 7-item Adolescent Psychotic-like Symptom Screener (APSS), and were interviewed by 2 psychiatrists using the Schedule for Affective Disorders and Schizophrenia for School-Aged Children (K-SADS), Present and Lifetime Versions.^39^ K-SADS is a well-validated and widely used tool in the assessment of current and lifetime Diagnostic and Statistical Manual of Mental Disorders, Fifth Edition (DSM-V) mental disorders in children and adolescents. A recent Portuguese study suggested the sensitivity, specificity, and accuracy of the K-SADS for psychotic phenomena in young adolescents as 88, 88, and 91, respectively.^40^ Participants who scored ≥ 2 points in the questionnaire indicated some degree of past experience of PEs. Those who scored less than 2 points were deemed controls. All participants, regardless of PE or control status had no formal diagnosis of any psychotic syndrome and were naïve for both antipsychotics and illicit substance use. The 95 who agreed to undergo MRI neuroimaging at baseline were invited to 2 follow-up neuroimaging sessions at around 2 and 5 years after baseline.

### MRI Acquisition

All scanning was performed on the same scanner (Philips Intera Achieva 3.0 Tesla) at the Trinity College Institute of Neuroscience, Dublin.^19^ A total of 180 axial high-resolution T1-weighted anatomical images (time in echo [TE] = 3.8 ms, repetition time [TR] = 8.4 ms, field of view [FOV]: 230 × 230 mm^2^, 0.898 × 0.898 mm^2^ in-plane resolution, slice thickness 0.9 mm, flip angle alpha = 8°) were acquired. The acquisition time was approximately 6.5 min. Diffusion scans were also acquired during the same session. Scans were checked for quality and de-identified for group, sex, and age before analysis.

### Freesurfer Analysis and Thalamic Nuclei Volume Estimation

Freesurfer is an automated image analysis suite for cortical reconstruction and volumetric segmentation (http://surfer.nmr.mgh.harvard.edu/).^41^ The technical details of the processing procedures are described elsewhere.^42^ For this study, the thalamus segmentation tool from Freesurfer (v7.4., 2023) was used to calculate thalamic nuclei volumes from the T1 images.^27^ This technique uses novel algorithms using Bayesian inference and ultra-high-resolution in-vivo and ex-vivo MRI reference template data to estimate 26 thalamic nuclei on each side. All nuclei volumes were rendered and checked visually for approximate shape, volume, and location within the thalamus for face validity prior to any analysis. Freesurfer also generated an estimated measure for total intracranial volume (eTIV) using the MRI-seg-stats tool.

### Statistical Analysis

To test the hypothesis, the data were imported into SPSS Statistics 28 [IBM, 2022]. Data were winsorized to account for outliers. Normality of the data was further assessed by using the Shapiro–Wilk test.

Mixed-method analyses of variance (ANOVAs) were used to investigate group-wise differences in volumes and across all nuclei in both left and right thalamic hemispheres.

Assumption testing included evaluating homogeneity of regression slopes and assessing equality of variances using Levene’s test. This allowed for correction of confounding variables such as age, sex, and total cross-sectional intracranial volume, and to identify any difference between control and PE groups.^41^ Partial eta-squared (η^2^_p_) values were calculated as a measure of effect size. Significance thresholds were corrected using the Benjamini–Hochberg method controlling the false discovery rate (FDR) using sequential modified Bonferroni correction for multiple hypothesis testing throughout.^42^ Modified FDR-corrected *P*-values were calculated for each subnucleus per timepoint in isolation. This resulted in *n* = 50 FDR-corrected *P*-values per TP, with *n* = 150 across the 3 TPs cumulatively.

As participants underwent MRI scans to profile thalamic subnuclear changes in the volume across time, a linear mixed-effects (LME) analysis was performed to capture the longitudinal nature of the data.^43^ Linear mixed-effects methods were chosen as they allow for profiling of the evolution of thalamic subnuclear volumetric changes as participants undergo MRI scans across follow-up timepoints with varying intervals of time between measurements. Linear mixed-effects methods allow for inclusion of participants with missing data points, and a combination of components of fixed effects, random effects, and repeated measures within a single model.^44^ The variables in this model included subject (random), age (fixed), and grouping (fixed). Scatter plots were also created for each thalamic subnucleus to observe for linearity of volumetric changes across timepoints. The interaction between age and grouping (slope) was used to interpret potential deviances in developmental trajectory, while the group differences showed effects at the level of the intercept. False discovery rate correction was similarly used for post-hoc evaluation of results. *n* = 50 FDR corrected *P*-values were calculated (one per subnucleus being examined).

### Demographics

At baseline, timepoint 1 (TP1), there were 53 participants in the PE group and 45 healthy controls (Table 1). Within the PE group, 39.6% were female (*n* = 21) and the mean age was 1 67.4 months (SD: ± 14.5). Controls consisted of 23 females (51.1%) with a mean age of 171.3 months (SD: ± 16.9). Chi-squared tests were performed to assess for statistical difference between characteristics of demographic groups at baseline with no significant differences between demographic groups. At timepoint 2 (TP2), there were 37 participants in the PE group and 31 healthy controls. Within the PE group, 45.9% were female (*n* = 17) and the mean age was 193.1 months (SD = 15.1). Controls consisted of 18 females (58.1%) with a mean age of 195.5 months (SD = 16.6). At timepoint 3 (TP3), there were 33 participants in the PE group and 21 healthy controls. Within the PE group, 30.3% were female (*n* = 10) and the mean age was 240.7 months (SD: ± 18.3). Controls consisted of 13 females (61.9%) with a mean age of 244 months (SD: ± 21.7).

**Table 1:** Demographic Data

	Timepoint 1		Timepoint 2		Timepoint 3	
	(*n* = 98)		(*n* = 68)		(*n* = 54)	
	PE	HC	PE	HC	PE	HC
	(*n* = 53)	(*n* = 45)	(*n* = 37)	(*n* = 31)	(*n* = 33)	(*n* = 21)
Return % group			69.8	68.9	89.2	67.8
Return % total			69.4		79.4	
Age, months (SD)	167.4 (14.5)	171.3 (16.9)	193.1 (15.1)	195.5 (16.6)	240.7 (18.3)	244 (21.7)
Gender (male/female)	32/21	22/23	20/17	13/18	23/10	8/13

Age and sex of young adolescents with PEs and controls at each timepoint. Abbreviations: HC, healthy controls; PE, psychotic experience; SD, standard deviation.

### Cross-sectional Analysis

A cross-sectional analysis of mean thalamic subnuclear volumes between PE and controls at each timepoint (TP1, TP2, and TP3) was performed (Tables S2–S4). A mixed-method ANOVA, correcting for age, sex, and eTIV was performed to compare thalamic subnuclei between the PE group and healthy controls for each TP in isolation (Table 2). At TP1, only the left lateral geniculate nucleus (*F* = 6.322, *P* = .014) was found to be significantly larger in the PE group compared to healthy controls. At TP2, a significant group effect was found for the left pulvinar intermediate (*F* = 9.782, *P* < .001), left pulvinar medial (PuM) (*F* = 4.445, *P* = .039), and left pulvinar anterior (PuA) (*F* = 4.071, *P* = .048) volumes, with all nuclei found to be significantly smaller in the PE group compared to healthy controls. However, at TP3, the left medial ventral (reunions) (*F* = 4.995, *P* = .03) and right paracentral (*F* = 6.392, *P* = .015) nuclei were found to be significantly larger in PE groups while the left pulvinar lateral (*F* = 4.871, *P* = .034) was found to be larger in healthy controls. As subnuclei within the pulvinar cluster were significant across several timepoints, volumes in the overall pulvinar composite were also examined cross-sectionally across timepoints. A significant group effect was found for the left pulvinar nucleus in both TP2 (*P* = .01) and TP3 (*P* = .019). Post-hoc analysis was applied via FDR method to correct for multiple comparisons. No results survived post-hoc correction.

**Table 2:** Cross-sectional Analysis Showing Significant Differences (*P* < .05) at Each Timepoint

Thalamic nuclei	*F*	Sig.	$\eta^{\text 2}_{\text p}$	FDR threshold
**Timepoint 1**				
LeftLGN	6.322	**0.014**	0.064	0.001
Left pulvinar composite	3.545	0.116	0.035	N/A
**Timepoint 2**				
LeftPuI	9.782	**0.00**	0.134	0.001
LeftPuM	4.445	**0.039**	0.066	0.002
LeftPuA	4.071	**0.048**	0.061	0.003
Left pulvinar composite	5.465	**0.01**	0.084	N/A
**Timepoint 3**				
LeftMVRe	4.995	**0.03**	0.093	0.002
LeftPuL	4.781	**0.034**	0.089	0.003
RightPc	6.392	**0.015**	0.115	0.001
Left pulvinar composite	5.364	**0.019**	0.098	N/A

ANCOVAs were performed to reveal differences between young adolescents with PEs and controls at each of the 3 timepoints. No nuclei reached the threshold for significance using strict FDR correction at any timepoint. However, using a *P*-value of less than .05, the nuclei in bold reached the threshold for trend level significance. As all 4 left pulvinar nuclei reached trend level significance at various timepoints, a composite left pulvinar group was created by summing the all pulvinar volumes. ANCOVAs for the pulvinar composite revealed differences between PEs and controls at TP2 and TP3 (left pulvinar TP2: *P* = .01, left pulvinar TP3: *P* = .019). Sig = *P-*value, η^2^_p_ = partial eta squared, FDR threshold = adjusted *P*-value when corrected using the Benjamini–Hochberg method controlling the FDR using sequential modified Bonferroni correction for multiple hypothesis testing. Abbreviations: ANCOVA, analysis of covariance; FDR, false discovery rate; LGN, lateral geniculate; MVRe, medial ventral reuniens; Pc, parcentral; PE, psychotic experience; PuA, pulvinar anterior; PuI, pulvinar inferior; PuL, pulvinar lateral; PuM, pulvinar medial.

### Longitudinal Analysis

To profile the change in thalamic subnuclear volumes in the PE versus control group longitudinally across timepoints, LME modeling with post-hoc FDR correction was applied. Significant group effect was observed for 2 left-sided ventral nuclei; the left ventral anterior magnocellular (VAmc) (*F* = 0.041, slope = 0.059, *P* = .041) and left ventral lateral anterior (VLa) (*F* = 0.002, slope = 0.005, *P* = .003), and 2 left-sided pulvinar nuclei; left PuA (*F* = 0.048, slope = 0.014, *P* = .048) and left PuM (*F* = 0.019, slope = 0.003, *P* = .019) (Table 3). However, none of these survived post-hoc correction (Table S5).

**Table 3:** Longitudinal Analysis Across 3 Timepoints Showing Significant Differences at *P* < .05

Significant LME results			
Nucleus	Group effect	FDR threshold	Slope
LeftPuA	0.048	0.006	0.014
LeftVAmc	0.041	0.004	0.059
LeftPuM	0.019	0.003	0.003
LeftVLa	0.003	0.002	0.001
LeftVA	0.002	0.001	0.005

The above nuclei reached the threshold for trend level significance (*P* < .05), indicated by the above group effect value. However, none of these values survived post-hoc correction. FDR threshold = adjusted *P*-value when corrected using the Benjamini–Hochberg method controlling the FDR using sequential modified Bonferroni correction for multiple hypothesis testing. Abbreviations: FDR, false discovery rate; LME, linear mixed effects; PuA, pulvinar anterior; PuM, pulvinar medial; VA, ventral anterior; VAmc, ventral anterior magnocellular; VLa, ventral lateral anterior.

As both multiple pulvinar and ventral nuclei approached significance, a further LME was performed on the pulvinar composite and a ventral composite. The ventral composite comprised of the ventral anterior, VAmc, VLa, ventral lateral posterior, ventral posterolateral, and ventromedial nuclei. The left pulvinar (*P* = .008) and left ventral (*P* = .013) composites remained significant after correction for multiple comparisons.

## Discussion

**Figure 1:**
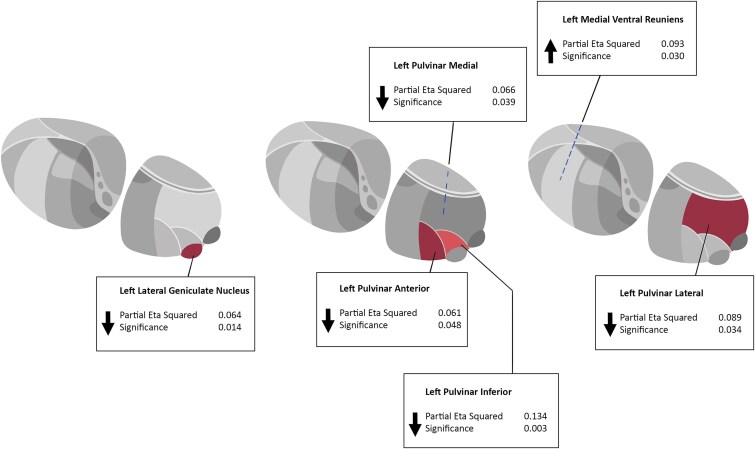
Showing the Individual Nuclei Reaching Trend Level (*P* < .05) But Not Strict FDR Significance at Timepoints 1, 2, and 3.

This study investigated volumetric differences in thalamic nuclei between adolescents with a history of PEs compared to healthy controls at and across 3 timepoints. No individual nucleus maintained a significant difference following strict testing for multiple comparisons at any timepoint. However, several pulvinar nuclei showed trend-level differences (ie, *P* < .05, but not reaching an FDR threshold) at TP2 and TP3 (Table 2; Figure 1). Reorganization of all the pulvinar nuclei into a combined pulvinar composite revealed a smaller left-sided pulvinar region overall in PEs at TP2 (*P* = .01) and TP3 (*P* = .019). Similarly, on longitudinal analysis, no nucleus maintained a significant difference following strict testing for multiple comparisons. Pulvinar and ventral composite reorganizations showed left-sided pulvinar region decreases (*P* = .008) and left-sided ventral increases (*P* = .013) over time.

### Pulvinar Regions

**Figure 2:**
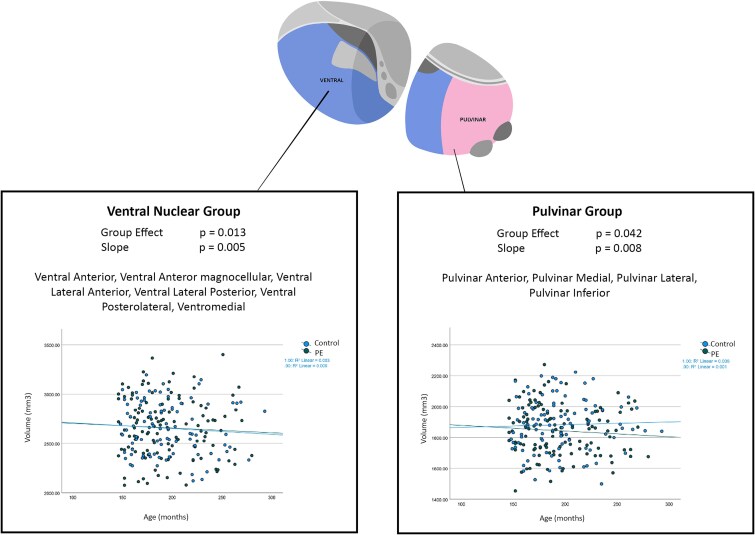
Showing the Composite Pulvinar and Ventral Nuclear Groups Reaching Significance With Linear Mixed-Effects Modeling Across the 3 Timepoints.

The left pulvinar composite region demonstrated a reduced volume at TP2 and TP3 and over time in adolescents with a history of PEs when compared with health controls (Table 3; Figure 2). These findings are substantiated by previous literature, showing a reduction in pulvinar volume in adolescents on the psychotic spectrum.^33^ Post-mortem studies have found smaller pulvinar volumes and neuron numbers in schizophrenia.^29^^,^^43^ The pulvinar is the largest nuclear group within the thalamus and, although classically associated with visual input processing,^34^ is also involved with general cognitive function specifically cognitive flexibility, synchronization and goal-directed attention.^35^ The thalamus is involved in modulating cortico-cortical communication via cortico-thalamocortical pathways,^35^ facilitating synchronization across cortical regions during tasks requiring attention.^44^ Pulvinar lesions have been shown to result in reduced attention signals transmitted to the cortex.^44^ The medial pulvinar, in particular, integrates visual information with higher-order cognitive and attentional functions through its extensive connections with parietal, temporal, and prefrontal cortices^45^ and its abnormal structure and connectivity have been linked to core features of psychosis, including impaired sensory integration and attentional control.^45^ A core deficit in psychosis may involve the inappropriate modulation of attention via dyssynchronous recruitment of cortical networks. Disrupted cortical synchronization via a smaller pulvinar region may be a cause for the reality testing difficulties found in psychosis and our young adolescents with PEs. Interestingly, previous studies show that pulvinar size can predict the level of cognition changes found in patients with psychosis.^46^ Reduced dopamine D2 receptor binding has also been found in the pulvinar of patients with psychosis^47^ with lower D2 receptor binding associated with more severe psychotic symptoms.^48^^,^^49^

### Ventral Regions

The left ventral composite region demonstrated a larger volume over time in adolescents in adolescents with a history of PEs when compared with health controls (Table 3; Figure 2). The ventral nuclear complex plays a central role in motor control. The ventral anterior, lateral, medial, and posterolateral nuclei are closely linked with the basal ganglia and receive input from both the globus pallidus and midbrain dopaminergic areas,^26^ and connect to the somatosensory, motor, and prefrontal cortices integrating motor, sensory, proprioceptive, and cognitive information.^50^^,^^51^ Ventral nuclei dysfunction can result in motor difficulties and reduced coordination.^52^ Similarly, motor impairments are common in psychotic spectrum disorders, with motor manifestations emerging early in premorbid and prodromal stages of the neurodevelopmental trajectory, often before overt or diagnosable psychotic disorders.^53^ Interestingly, a parallel cohort from the ABD study of young adolescents with similar PEs revealed fine motor coordination difficulties compared to controls.^54^ The ventral nuclei and midbrain dopaminergic neuron connections are intriguing due to the integral role of dopaminergic signaling in psychosis.^3^ Abnormalities in dopamine signaling have long been associated with the underlying pathogenesis of psychosis, with the establishment of the dopaminergic hypothesis of schizophrenia in the 1950s^55^ excessive drug-induced D2 receptor stimulation known to cause positive psychotic symptoms^56^ and D2 blockade being the mainstay of pharmacological antipsychotic treatment. The larger ventral nuclei in our PE group hint at a potential anatomical locus integrating dopaminergic involvement in developing PEs. The observed increase in ventral thalamic volume may reflect a neurodevelopmental or compensatory response in a system influenced by persistent dopaminergic input. Emerging models suggest that psychosis-related dopamine overactivity may result from upstream dysregulation, particularly from the ventral hippocampus, rather than intrinsic abnormalities in the dopamine system itself.^57^ Rodent models show that hippocampal hyperactivity, potentially due to reduced GABAergic inhibition, can drive increased dopaminergic neuron firing via a circuit involving the nucleus accumbens, ventral pallidum, and possibly the ventral thalamus (ref). Thus, increased ventral thalamic volume in the PE group may mark a structural correlate of this dysregulated circuit, contributing to dopaminergic hyper-responsivity and the aberrant salience attribution characteristic of psychosis.^58^

### Strengths and Limitations

The strengths of this study include a well-described and well-matched sample of medication and substance-naive adolescents. The use of a community-based sample rather than a hospital or clinic-based sample increases the generalizability of these results to other adolescent groups. A total of 70% of the sample returned for repeat scanning at T2, with 80% of those (55% of the original cohort) returning at T3 (Table 1). This is an acceptable return rate for a nonclinical, originally school-going sample of young adolescents over 5 years, as they progress into adulthood and often get lost to follow-up. High resolution of the T1 imaging allows for accurate delineation of thalamic nuclei using a state-of-the-art parcellation technique and the automated nature of the parcellation, increases the reliability and removes individual rater biases often found in manual parcellation.

In terms of limitations, we acknowledge the relatively small, Irish-only sample and that our findings require replication in a larger and broader international sample. Automated segmentation has been argued to have less interpretative and validity precision compared with expert manual segmentation.^59^^,^^60^ Different automated thalamic protocols exist (not just Freesurfer-based) using different parcellation methods. Such protocols can differ considerably from one another in terms of substructure terminology, defined regional boundaries, and segmentation protocols. Such discrepancies may be particularly evident for smaller thalamic substructures possibly reducing the validity of between study interpretations.

## Conclusion

Using advanced automated thalamic nuclei parcellation of high-resolution structural MRI, this study demonstrates smaller left-sided pulvinar and larger ventral thalamic region volumes in adolescents with PEs when compared with controls from the ages of around 11-13 to 19-21 years old. It is noted that this is a nonclinical sample, with no individual meeting the threshold for a formal diagnosis of psychotic disorder or even presenting to healthcare providers with symptoms. Such findings in a nonclinical sample of young adolescents with low-level PEs suggest that thalamic changes relating to attention, cortical synchronization, and dopaminergic tone may occur earlier along the spectrum of psychosis than previously thought. These results reflect similar but more profound changes in the thalamus (in particular the pulvinar region) in higher-risk individuals and those with psychotic disorders. Thalamic circuitry perturbations occurring early along the psychosis spectrum may be a potential vulnerability marker for the development of clinical psychosis.

## Supplementary Material

Supplemental_sgaf016
