# Supplementary material for: Pulvinar and Ventral Thalamic Nuclei Changes Occur Early Along the Psychosis Spectrum
Source: Schizophr Bull Open. 2025 Sep 11;6(1):sgaf016. doi: 10.1093/schizbullopen/sgaf016 (PMC12476835; doi:10.1093/schizbullopen/sgaf016)
Supplement: Supplemental_sgaf016 [file supplemental_sgaf016.docx]

| **Group** | **Abbreviation** | **Nucleus** | **Function** |
| --- | --- | --- | --- |
| **Anterior** | AV | Anteroventral | These nuclei are thought to play a role in the modulation of alertness and are involved in learning and episodic memory. They are considered to be part of the limbic system. |
| **Lateral** | LD | Laterodorsal | These nuclei are considered to be involved in spatial learning and memory and the integration and modulation of visual, somatosensory and motor signals. |
|  | LP | Lateral posterior |  |
| **Ventral** | VA | Ventral anterior | These nuclei communicate with the basal ganglia and cerebellum, forming a thalamostriatal network, with various functions, including motor control, sensory processing, and limbic system modulation |
|  | Vamc | Ventral Anterior magnocellular |  |
|  | VLa | Ventral lateral anterior |  |
|  | VLp | Ventral lateral posterior |  |
|  | VPL | Ventral posterolateral |  |
|  | VM | Ventromedial |  |
| **Intralaminar** | CeM | Central medial | These nuclei play a role in the activation of the cortex from the  brainstem reticular formation. They also involved in sensory-motor integration. |
|  | CL | Central lateral |  |
|  | Pc | Paracentral |  |
|  | CM | Centromedian |  |
|  | Pf | Parafascicular |  |
| **Medial** | Pt | Paratenial | These nuclei are involved in a range of functions, including emotional processing, memory, and regulation of certain visceral and autonomic activities |
|  | MVRe | Reuniens (medial ventral) |  |
|  | MDm | Mediodorsal medial magnocellular |  |
|  | MDl | Mediodorsal lateral parvocellular |  |
| **Posterior** | LGN | Lateral geniculate | These nuclei play important roles in sensory processing,  particularly related to visual and auditory information. The pulvinar nuclei in particular play a role in visual information processing |
|  | MGN | Medial geniculate |  |
|  | LSG | Limitans (suprageniculate) |  |
|  | PuA | Pulvinar anterior |  |
|  | PuM | Pulvinar medial |  |
|  | PuL | Pulvinar lateral |  |
|  | PuI | Pulvinar inferior |  |

**Supplementary Table 1.** The 25 Freesurfer thalamic nuclei, functional composite nuclear groupings and possible functions.

| **Timepoint 1** | | | | |
| --- | --- | --- | --- | --- |
| **Thalamic Nuclei** | **F** | **Sig** | **η^2^_p_** | **FDR threshold** |
| LeftMGN | 2.314 | 0.132 | 0.024 | 0.026 |
| LeftLGN | 6.322 | 0.014* | 0.064 | 0.001 |
| LeftPuI | 1.348 | 0.249 | 0.014 | 0.007 |
| LeftPuM | 0.001 | 0.972 | 0 | 0.049 |
| LeftLSg | 0.031 | 0.86 | 0 | 0.044 |
| LeftVPL | 0.055 | 0.814 | 0.001 | 0.042 |
| LeftCM | 0.001 | 0.973 | 0 | 0.05 |
| LeftVLa | 0.439 | 0.509 | 0.005 | 0.028 |
| LeftPuA | 0.015 | 0.904 | 0 | 0.046 |
| LeftMDm | 0.244 | 0.622 | 0.003 | 0.035 |
| LeftPf | 0.493 | 0.485 | 0.005 | 0.027 |
| LeftVAmc | 2.847 | 0.095 | 0.03 | 0.003 |
| LeftMDl | 1.101 | 0.297 | 0.012 | 0.014 |
| LeftCeM | 2.101 | 0.151 | 0.022 | 0.008 |
| LeftVA | 3.732 | 0.056 | 0.039 | 0.002 |
| LeftMVRe | 2.429 | 0.123 | 0.025 | 0.006 |
| LeftVM | 0.569 | 0.452 | 0.006 | 0.025 |
| LeftCL | 0.795 | 0.375 | 0.008 | 0.019 |
| LeftPuL | 0.754 | 0.387 | 0.008 | 0.02 |
| LeftPt | 0.266 | 0.607 | 0.003 | 0.034 |
| LeftAV | 1.25 | 0.266 | 0.013 | 0.011 |
| LeftPc | 1.124 | 0.292 | 0.012 | 0.013 |
| LeftVLp | 0.325 | 0.57 | 0.003 | 0.031 |
| LeftLP | 2.632 | 0.108 | 0.028 | 0.004 |
| LeftLD | 0.985 | 0.323 | 0.01 | 0.018 |
| RightLGN | 0.73 | 0.395 | 0.008 | 0.001 |
| RightMGN | 0.52 | 0.473 | 0.006 | 0.007 |
| RightPuI | 1.516 | 0.221 | 0.016 | 0.01 |
| RightPuM | 0.219 | 0.641 | 0.002 | 0.036 |
| RightLSg | 0.388 | 0.535 | 0.004 | 0.029 |
| RightVPL | 0.625 | 0.431 | 0.007 | 0.024 |
| RightCM | 1.055 | 0.307 | 0.011 | 0.016 |
| RightVLa | 0.105 | 0.747 | 0.001 | 0.04 |
| RightPuA | 0.311 | 0.579 | 0.003 | 0.032 |
| RightMDm | 0.004 | 0.947 | 0 | 0.048 |
| RightPf | 0.347 | 0.557 | 0.004 | 0.03 |
| RightVAmc | 1.049 | 0.308 | 0.011 | 0.017 |
| RightMDl | 1.082 | 0.301 | 0.012 | 0.015 |
| RightVA | 0.035 | 0.852 | 0 | 0.043 |
| RightMVRe | 0.005 | 0.945 | 0 | 0.047 |
| RightCeM | 0.102 | 0.751 | 0.001 | 0.041 |
| RightVM | 0.127 | 0.723 | 0.001 | 0.038 |
| RightPuL | 0.659 | 0.419 | 0.007 | 0.023 |
| RightCL | 0.703 | 0.404 | 0.008 | 0.022 |
| RightVLp | 0.169 | 0.682 | 0.002 | 0.037 |
| RightPc | 0.302 | 0.584 | 0.003 | 0.033 |
| RightPt | 0.11 | 0.741 | 0.001 | 0.039 |
| RightAV | 0.026 | 0.871 | 0 | 0.045 |
| RightLP | 1.214 | 0.273 | 0.013 | 0.012 |
| RightLD | 2.443 | 0.121 | 0.026 | 0.005 |

**Supplementary Table 2. Cross-sectional analysis showing differences at timepoint 1**

ANCOVA results for full 50 (25 left, 25 right) nuclei at timepoint 1. Covariates included age, sex and eTIV. No nuclei reached threshold for significance following FDR correction. Nuclei that reached trend level effects (p<0.05) but not significance with FDR are identified with an asterisk. ANCOVA, analysis of covariance; eTIV, estimated total intracranial volume; FDR, false discovery rate. Nuclear abbreviations are found in Supplementary Table 1. Sig = p-value, η^2^_p_ = partial eta squared, FDR threshold = adjusted p-value when corrected using the Benjamini–Hochberg method controlling the False Discovery Rate (FDR) using sequential modified Bonferroni correction for multiple hypothesis testing

| **Timepoint 2** | | | | |
| --- | --- | --- | --- | --- |
| **Thalamic Nuclei** | **F** | **Sig.** | **η^2^_p_** | **FDR Threshold** |
| LeftMGN | 0.058 | 0.81 | 0.001 | 0.045 |
| LeftLGN | 3.22 | 0.078* | 0.049 | 0.008 |
| LeftPuI | 9.782 | 0.003* | 0.134 | 0.001 |
| LeftPuM | 4.445 | 0.039* | 0.066 | 0.002 |
| LeftLSg | 0.716 | 0.401 | 0.011 | 0.028 |
| LeftVPL | 0.731 | 0.396 | 0.011 | 0.027 |
| LeftCM | 3.248 | 0.076 | 0.049 | 0.007 |
| LeftVLa | 0.158 | 0.692 | 0.003 | 0.041 |
| LeftPuA | 4.071 | 0.048* | 0.061 | 0.003 |
| LeftMDm | 0.129 | 0.721 | 0.002 | 0.043 |
| LeftPf | 1.197 | 0.278 | 0.019 | 0.015 |
| LeftVAmc | 0.995 | 0.322 | 0.016 | 0.017 |
| LeftMDl | 0.015 | 0.903 | 0 | 0.048 |
| LeftCeM | 0.021 | 0.886 | 0 | 0.047 |
| LeftVA | 0.396 | 0.532 | 0.006 | 0.032 |
| LeftMVRe | 0.003 | 0.959 | 0 | 0.024 |
| LeftVM | 0.893 | 0.348 | 0.014 | 0.019 |
| LeftCL | 0.846 | 0.361 | 0.013 | 0.022 |
| LeftPuL | 1.733 | 0.193 | 0.027 | 0.012 |
| LeftPt | 0.856 | 0.358 | 0.013 | 0.021 |
| LeftAV | 0.122 | 0.728 | 0.002 | 0.044 |
| LeftPc | 3.26 | 0.076 | 0.049 | 0.006 |
| LeftVLp | 0.457 | 0.501 | 0.007 | 0.03 |
| LeftLP | 0.233 | 0.631 | 0.004 | 0.036 |
| LeftLD | 0.222 | 0.639 | 0.004 | 0.037 |
| RightLGN | 3.195 | 0.079 | 0.048 | 0.009 |
| RightMGN | 0.295 | 0.589 | 0.005 | 0.033 |
| RightPuI | 0.763 | 0.386 | 0.012 | 0.025 |
| RightPuM | 0.182 | 0.671 | 0.003 | 0.039 |
| RightLSg | 1.373 | 0.246 | 0.021 | 0.014 |
| RightVPL | 1.121 | 0.294 | 0.017 | 0.016 |
| RightCM | 0.207 | 0.651 | 0.003 | 0.038 |
| RightVLa | 0.746 | 0.391 | 0.012 | 0.026 |
| RightPuA | 0.444 | 0.508 | 0.007 | 0.031 |
| RightMDm | 0.039 | 0.843 | 0.001 | 0.046 |
| RightPf | 0.496 | 0.484 | 0.008 | 0.029 |
| RightVAmc | 0.151 | 0.699 | 0.002 | 0.042 |
| RightMDl | 0.25 | 0.619 | 0.004 | 0.034 |
| RightVA | 0.177 | 0.675 | 0.003 | 0.04 |
| RightMVRe | 0.795 | 0.376 | 0.012 | 0.024 |
| RightCeM | 0.924 | 0.34 | 0.014 | 0.018 |
| RightVM | 0.872 | 0.354 | 0.014 | 0.02 |
| RightPuL | 0.804 | 0.373 | 0.013 | 0.023 |
| RightCL | 0.233 | 0.631 | 0.004 | 0.035 |
| RightVLp | 2.062 | 0.156 | 0.032 | 0.01 |
| RightPc | 1.839 | 0.18 | 0.028 | 0.011 |
| RightPt | 0.002 | 0.965 | 0 | 0.05 |
| RightAV | 1.664 | 0.202 | 0.026 | 0.013 |
| RightLP | 3.588 | 0.063 | 0.054 | 0.004 |
| RightLD | 3.334 | 0.073 | 0.05 | 0.005 |
|  |  |  |  |  |

**Supplementary Table 3. Cross-sectional analysis showing differences at timepoint 2**

ANCOVA results for full 50 (25 left, 25 right) nuclei at timepoint 2. Covariates included age, sex and eTIV. No nuclei reached threshold for significance following FDR correction. Nuclei that reached trend level effects (p<0.05) but not significance with FDR are identified with an asterisk. ANCOVA, analysis of covariance; eTIV, estimated total intracranial volume. Nuclear abbreviations are found in Supplementary Table 1. Sig = p-value, η^2^_p_ = partial eta squared, FDR threshold = adjusted p-value when corrected using the Benjamini–Hochberg method controlling the False Discovery Rate (FDR) using sequential modified Bonferroni correction for multiple hypothesis testing

| **Timepoint 3** | | | | |
| --- | --- | --- | --- | --- |
| **Dependent Variable** | **F** | **Sig.** | **η^2^_p_** | **FDR** |
| LeftMGN | 1.601 | 0.212 | 0.032 | 0.006 |
| LeftLGN | 3.563 | 0.065 | 0.068 | 0.016 |
| LeftPuI | 3.346 | 0.073 | 0.064 | 0.007 |
| LeftPuM | 3.554 | 0.065 | 0.068 | 0.005 |
| LeftLSg | 0.231 | 0.633 | 0.005 | 0.033 |
| LeftVPL | 0.044 | 0.834 | 0.001 | 0.045 |
| LeftCM | 1.816 | 0.184 | 0.036 | 0.015 |
| LeftVLa | 0.245 | 0.623 | 0.005 | 0.03 |
| LeftPuA | 2.09 | 0.155 | 0.041 | 0.014 |
| LeftMDm | 0.324 | 0.572 | 0.007 | 0.028 |
| LeftPf | 0.058 | 0.811 | 0.001 | 0.041 |
| LeftVAmc | 0.598 | 0.443 | 0.012 | 0.024 |
| LeftMDl | 0.892 | 0.35 | 0.018 | 0.022 |
| LeftCeM | 3.61 | 0.063 | 0.069 | 0.004 |
| LeftVA | 1.379 | 0.246 | 0.027 | 0.017 |
| LeftMVRe | 4.995 | 0.03* | 0.093 | 0.002 |
| LeftVM | 0.016 | 0.901 | 0 | 0.048 |
| LeftCL | 0.026 | 0.874 | 0.001 | 0.047 |
| LeftPuL | 4.781 | 0.034* | 0.089 | 0.003 |
| LeftPt | 1.263 | 0.266 | 0.025 | 0.018 |
| LeftAV | 0.24 | 0.627 | 0.005 | 0.031 |
| LeftPc | 2.427 | 0.126 | 0.047 | 0.012 |
| LeftVLp | 0.223 | 0.639 | 0.005 | 0.035 |
| LeftLP | 0.21 | 0.649 | 0.004 | 0.036 |
| LeftLD | 0.143 | 0.707 | 0.003 | 0.038 |
| RightLGN | 1.216 | 0.276 | 0.024 | 0.019 |
| RightMGN | 1.025 | 0.316 | 0.02 | 0.021 |
| RightPuI | 2.539 | 0.118 | 0.049 | 0.011 |
| RightPuM | 0.395 | 0.533 | 0.008 | 0.025 |
| RightLSg | 0.299 | 0.587 | 0.006 | 0.029 |
| RightVPL | 0.049 | 0.826 | 0.001 | 0.044 |
| RightCM | 0.234 | 0.63 | 0.005 | 0.032 |
| RightVLa | 0.039 | 0.843 | 0.001 | 0.046 |
| RightPuA | 2.102 | 0.153 | 0.041 | 0.013 |
| RightMDm | 0.228 | 0.635 | 0.005 | 0.034 |
| RightPf | 0.1 | 0.753 | 0.002 | 0.04 |
| RightVAmc | 1.087 | 0.302 | 0.022 | 0.02 |
| RightMDl | 0.129 | 0.721 | 0.003 | 0.039 |
| RightVA | 0.362 | 0.55 | 0.007 | 0.027 |
| RightMVRe | 2.814 | 0.1 | 0.054 | 0.009 |
| RightCeM | 3.095 | 0.085 | 0.059 | 0.008 |
| RightVM | 0.645 | 0.426 | 0.013 | 0.023 |
| RightPuL | 0.372 | 0.544 | 0.008 | 0.026 |
| RightCL | 0.049 | 0.825 | 0.001 | 0.042 |
| RightVLp | 0.165 | 0.686 | 0.003 | 0.037 |
| RightPc | 6.39 | 0.015* | 0.115 | 0.001 |
| RightPt | 2.54 | 0.117 | 0.049 | 0.01 |
| RightAV | 0.005 | 0.944 | 0 | 0.05 |
| RightLP | 0.049 | 0.826 | 0.001 | 0.043 |
| RightLD | 0.007 | 0.934 | 0 | 0.049 |

**Supplementary Table 4. Cross-sectional analysis showing differences at timepoint 3**

ANCOVA results for full 50 (25 left, 25 right) nuclei at timepoint 3. Covariates included age, sex and eTIV. No nuclei reached threshold for significance following FDR correction. Nuclei that reached trend level effects (p<0.05) but not significance with FDR are identified with an asterisk. ANCOVA, analysis of covariance; eTIV, estimated total intracranial volume; FDR, false discovery rate. Nuclear abbreviations are found in Supplementary Table 1.

| **All LME results** | | | |
| --- | --- | --- | --- |
| **Nucleus** | **Group Effect** | **Slope** | **FDR** |
| **LeftMGN** | 0.065 | 0.055 | 0.005 |
| **LeftLGN** | 0.31 | 0.779 | 0.024 |
| **LeftPuI** | 0.894 | 0.566 | 0.048 |
| **LeftPuM** | 0.019* | 0.003 | 0.003 |
| **LeftLSg** | 0.145 | 0.141 | 0.01 |
| **LeftVPL** | 0.355 | 0.191 | 0.026 |
| **LeftCM** | 0.797 | 0.609 | 0.042 |
| **LeftVLa** | 0.003* | 0.001 | 0.002 |
| **LeftPuA** | 0.048* | 0.014 | 0.006 |
| **LeftMDm** | 0.303 | 0.14 | 0.023 |
| **LeftPf** | 0.224 | 0.272 | 0.018 |
| **LeftVAmc** | 0.041* | 0.059 | 0.004 |
| **LeftMDl** | 0.68 | 0.337 | 0.039 |
| **LeftCeM** | 0.285 | 0.498 | 0.022 |
| **LeftVA** | **0.002** | 0.005 | 0.002 |
| **LeftMVRe** | 0.106 | 0.223 | 0.008 |
| **LeftVM** | 0.271 | 0.205 | 0.021 |
| **LeftCL** | 0.079 | 0.084 | 0.007 |
| **LeftPuL** | 0.555 | 0.277 | 0.035 |
| **LeftPt** | 0.181 | 0.215 | 0.014 |
| **LeftAV** | 0.183 | 0.249 | 0.016 |
| **LeftPc** | 0.525 | 0.577 | 0.034 |
| **LeftVLp** | 0.156 | 0.056 | 0.012 |
| **LeftLP** | 0.607 | 0.867 | 0.036 |
| **LeftLD** | 0.713 | 0.876 | 0.04 |
| **RightLGN** | 0.659 | 0.986 | 0.038 |
| **RightMGN** | 0.834 | 0.981 | 0.044 |
| **RightPuI** | 0.833 | 0.465 | 0.043 |
| **RightPuM** | 0.475 | 0.312 | 0.032 |
| **RightLSg** | 0.228 | 0.353 | 0.019 |
| **RightVPL** | 0.928 | 0.763 | 0.049 |
| **RightCM** | 0.149 | 0.185 | 0.011 |
| **RightVLa** | 0.492 | 0.31 | 0.033 |
| **RightPuA** | 0.182 | 0.082 | 0.015 |
| **RightMDm** | 0.143 | 0.087 | 0.009 |
| **RightPf** | 0.157 | 0.181 | 0.013 |
| **RightVAmc** | 0.198 | 0.219 | 0.017 |
| **RightMDl** | 0.88 | 0.561 | 0.046 |
| **RightVA** | 0.472 | 0.381 | 0.031 |
| **RightMVRe** | 0.982 | 0.951 | 0.05 |
| **RightCeM** | 0.894 | 0.807 | 0.047 |
| **RightVM** | 0.635 | 0.437 | 0.037 |
| **RightPuL** | 0.465 | 0.3 | 0.03 |
| **RightCL** | 0.337 | 0.4 | 0.025 |
| **RightVLp** | 0.867 | 0.61 | 0.045 |
| **RightPc** | 0.445 | 0.331 | 0.029 |
| **RightPt** | 0.36 | 0.371 | 0.027 |
| **RightAV** | 0.243 | 0.177 | 0.02 |
| **RightLP** | 0.747 | 0.96 | 0.041 |
| **RightLD** | 0.384 | 0.607 | 0.028 |

**Supplementary Table 5. Full longitudinal analysis across three timepoints.**

LME results for full 50 nuclei (25 left, 25 right) over three timepoints. Only the left ventral anterior nucleus reached the threshold for significance after FDR correction. Nuclei that reached trend level effects (p<0.05) but not significance with FDR are identified with an asterisk. FDR, false discovery rate; LME, linear mixed effects modelling. Nuclear abbreviations are found in Supplementary Table 1.
